# Enhanced Minor Ginsenoside Contents of Nano-Sized Black Korean Ginseng through Hot Melt Extrusion

**DOI:** 10.3390/ma17184612

**Published:** 2024-09-20

**Authors:** Junho Lee, Ha-Yeon Lee, Jong-Suep Baek

**Affiliations:** 1 Department of Bio-Health Convergence, Kangwon National University, Chuncheon 24341, Republic of Korea; junho9763@kangwon.ac.kr (J.L.); lhy101625@kangwon.ac.kr (H.-Y.L.); 2 BeNatureBioLab, Chuncheon 24206, Republic of Korea

**Keywords:** black ginseng, major ginsenosides, minor ginsenosides, ginsenoside Rg3, compound K, hot melt extrusion

## Abstract

Black ginseng (BG), a traditional medicinal herb produced through a nine-stage steaming and drying process, exhibits stronger pharmacological efficacy, including antioxidant, anti-inflammatory, and anti-cancer properties, when compared to white and red ginseng. The ginsenosides in BG are classified as major and minor types, with minor ginsenosides demonstrating superior pharmacological properties. However, their low concentrations limit their availability for research and clinical applications. In this study, hot melt extrusion (HME) was utilized as an additional processing technique to enhance the content of minor ginsenoside in BG, and the physicochemical properties of the formulation were analyzed. Ginsenoside content in BG and HME-treated BG (HME-BG) was analyzed using high-performance liquid chromatography (HPLC), while their physicochemical properties were evaluated through dynamic light scattering (DLS), electrophoretic light scattering (ELS), X-ray diffraction (XRD), thermogravimetric analysis (TGA), and Fourier-transform infrared spectroscopy (FT-IR). HME treatment resulted in a significant increase in minor ginsenosides Rg3 and compound K (CK) by 330% and 450%, respectively, while major ginsenosides Rg1 and Rb1 decreased or were not detected. Additionally, HME-BG demonstrated reduced particle size, improved PDI, and decreased crystallinity. HME treatment effectively converts major ginsenosides in BG into minor ginsenosides, enhancing its pharmacological efficacy and showing great potential for research and development applications.

## 1. Introduction

Ginseng, often referred to as the “king of herbs”, is a globally recognized supplement and tonic with a long history of use in traditional medicine [1]. This herb is processed into various forms, including white ginseng, red ginseng, and black ginseng, which serve as key ingredients in numerous functional foods. Among these, black ginseng is distinguished by a unique processing method that involves nine cycles of steaming and drying, a practice rooted in ancient Oriental medicine [2]. This meticulous process is believed to enhance the medicinal properties of black ginseng, which is known to exhibit more potent pharmacological effects compared to other ginseng forms, including improved immune function, antioxidant activity [3], anti-inflammatory [4], and anticancer properties [5,6].

The pharmacological efficacy of black ginseng is primarily attributed to its ginsenosides, which are categorized into major and minor types. Major ginsenosides, such as Rg1, Rb1, Rb2, and Rc, are the principal compounds responsible for ginseng’s therapeutic effects. In contrast, minor ginsenosides, including Rg3, Rg5, and compound K (CK), though present in lower concentrations, are noted for their superior pharmacological activities [7,8,9]. However, the naturally low abundance of these minor ginsenosides in plants poses challenges for scientific research and clinical application. While black ginseng contains a higher proportion of minor ginsenosides compared to white and red ginseng, their levels remain relatively low, typically around 1–2%. Therefore, developing processing techniques to further enhance the content of minor ginsenosides is crucial [10,11,12].

To address the challenge of enhancing the concentration of minor ginsenosides, various strategies have been explored, including physical treatments (drying, heat, microwave, etc.), chemical treatments (acid and base hydrolysis), and biological treatments (enzymes and microorganisms) to process ginseng into black ginseng [13,14,15]. Despite these efforts, issues such as limited conversion efficiency, quality degradation, residual chemicals, high costs, and process complexity remain significant obstacles.

Hot melt extrusion (HME) is a widely used technology in the pharmaceutical, food, and natural product industries, known for its ability to form solid dispersions that improve solubility and bioavailability [16,17,18]. HME has been shown to enhance the active ingredients as well as the antifungal, antibacterial, and anti-inflammatory properties of natural products such as *Angelica gigas Nakai* [16], mulberry leaves [19], and black mulberry [20]. Unlike conventional processing methods, such as solvent extraction that primarily uses organic solvents, supercritical extraction which incurs high costs, or fermentation that requires long-term maintenance of specific environmental conditions [21], HME offers several advantages. These include HME being an environmentally friendly process that does not require organic solvents and has a shorter processing time and greater ease of optimization due to its simplicity [16]. However, the application of HME technology to increase the content of minor ginsenosides in black ginseng has not yet been investigated.

This study aimed to enhance the concentration of minor ginsenosides, the primary active compounds in black ginseng, by structurally converting major ginsenosides using HME technology. Black ginseng powder treated with hot melt extrusion (HME-BG) was produced, and the levels of ginsenosides Rg1, Rb1, Rg3, and CK were compared with those in untreated black ginseng powder (BG) using HPLC analysis. Additionally, the particle properties of HME-BG were assessed.

## 2. Materials and Methods

### 2.1. Materials

Black ginseng powder (BG) was purchased from Guan Industry (Seoul, Republic of Korea). Ginsenoside Rg1 was purchased from TCI (Tokyo, Japan), Rb1 was purchased from PhytoLab GmbH & Co. KG (Vestenbergsgreuth, Germany), and Rg3 and CK were purchased from Sigma-Aldrich (St. Louis, MO, USA). All chemicals were of analytical grade.

### 2.2. Preparation of Black Ginseng Nanocapsules 

Black ginseng powder was fed into a co-rotating intermeshing type twin-screw extruder (STS25-28FD, Pyeongtaek, Republic of Korea) without any excipients. The extrusion hole had a 1 mm diameter circle. The manufacturing process of HME-BG was conducted at a temperature of 110 °C, a screw speed of 180 rpm, and a pressure of 15 bar using the HME equipment. Approximately 60 g of the sample was supplied per minute.

After the process was completed, the extrudate was dried at 70 °C for 12 h in an oven (SCOV-150, Sungchan Science, Pocheon, Republic of Korea), and then ground for use in the experiment and for storage.

### 2.3. Extraction of Ginsenosides

Extraction of ginsenosides from BG and HME-BG was carried out using 70% (*v*/*v*) ethanol. First, 1 g of sample powder was combined with 50 mL of 70% (*v*/*v*) ethanol and processed for 30 min at 40% amplitude in a pulsed mode (15 min on, 15 min off) using an ultrasonic processor (VCX130, Sonics & Materials Inc., Newtown, CT, USA). To enhance extraction efficiency, sonication was extended by an additional 30 min in a water bath. Following sonication, the mixture was centrifuged at 1509× *g* and filtered through a Whatman filter paper (No. 6). The extracts were prepared in triplicate and stored at 4 °C until analysis.

### 2.4. High-Performance Liquid Chromatography Analysis

All samples were filtered using a hydrophobic syringe filter (0.45 μm) before HPLC analysis. The equipment used was a 1200 series system with a diode array detector (DAD; Agilent Technologies, Waldbronn, Germany), and ginsenoside analysis was performed according to the method of Hoang et al. [1] with some modifications, as detailed in Table 1. Following the analysis, the quantities of ginsenosides Rg1, Rb1, Rg3, and Compound K were determined per 1 g of sample.

### 2.5. Measurement of Particle Size, Polydispersity Index, and Zeta Potential

The particle size, polydispersity index (PDI), and zeta potential (ZP) of the sample were measured using dynamic light scattering (DLS) and electrophoretic light scattering (ELS) with a ZSP device (Malvern Instruments, Malvern, UK). For the measurement, the sample was mixed in distilled water at a concentration of 1 mg/mL, dispersed in an Ultrasonic Cleaner (UCP-20, Jeio Tech, Daejeon, Republic of Korea) for 30 min, sedimented for 10 min, and the supernatant was collected for measurement.

### 2.6. X-ray Diffraction Analysis

An X-ray diffractometer (D8 Discover +, Bruker AXS GmbH, Billerica, MA, USA) was used to determine the crystallinity of the samples. The samples were placed on the sample stage of the instrument, and diffractograms were generated using Cu Kα radiation (Ni filter, generator setting: 50 kV) in Bragg–Brentano θ: 2θ geometry. The scan range was set to 5–80°, and the scanning step size was 0.03°.

### 2.7. Thermogravimetric Analysis

A simultaneous DSC-TGA analyzer (SDT650, TA Instruments, New Castle, DE, USA) was used for thermogravimetric analysis of the samples. Approximately 30 mg of the sample was placed on an alumina pan and heated from 20 to 700 °C at a rate of 10 °C min^−1^.

### 2.8. Fourier-Transform Infrared Spectroscopy

An infrared spectrometer (iS50, Thermo Scientific, Waltham, MA, USA) was used to track the changes in the properties of the samples. The samples were analyzed in the spectral range of 400–4000 cm^−1^ using Attenuated Total Reflectance (ATR).

### 2.9. Statistical Analysis

Data were measured in triplicate and are presented as mean and standard deviation (SD). Statistical significance was determined using one-way ANOVA, and a *p*-value of <0.05 was considered statistically significant. The analyses were conducted using PASW Statistics 18 (SPSS Inc., Chicago, IL, USA).

## 3. Results and Discussion

### 3.1. HPLC Analysis of Ginsenoside Content

**Figure 1:**
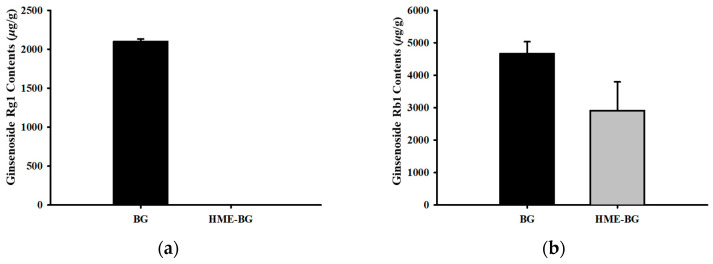

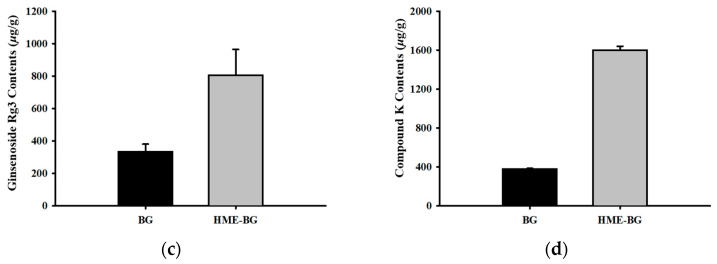
Comparison of ginsenoside contents in black ginseng powder (BG) and HME-treated black ginseng powder (HME-BG): (**a**) Ginsenoside Rg1; (**b**) Ginsenoside Rb1; (**c**) Ginsenoside Rg3; (**d**) Compound K.

**Figure 2:**
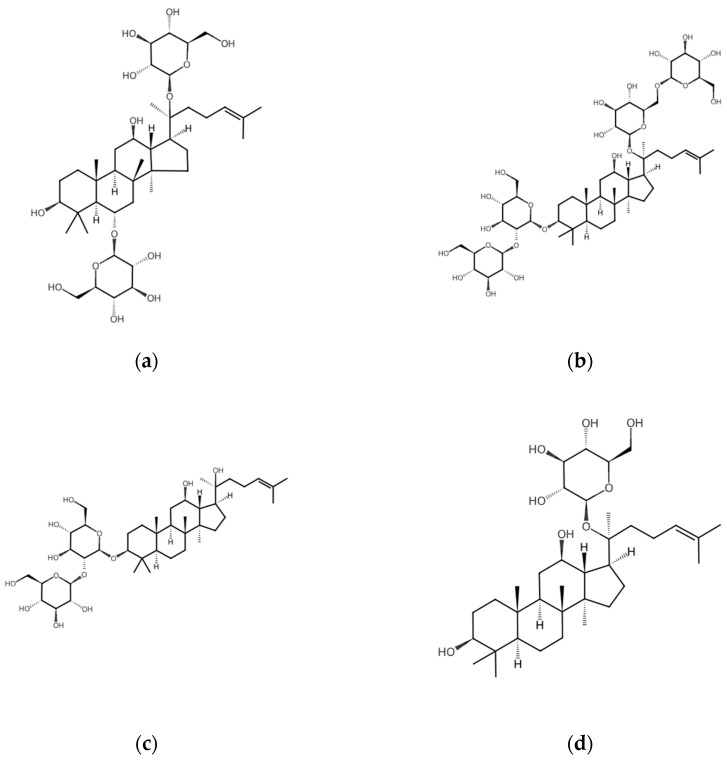
Chemical structures of ginsenosides: (**a**) Ginsenoside Rg1; (**b**) Ginsenoside Rb1; (**c**) Ginsenoside Rg3; (**d**) Compound K.

To examine the changes in the overall ginsenoside profile between BG and HME-BG, both were extracted using 70% (*v*/*v*) ethanol. The extracts were analyzed for the content of major ginsenosides, such as ginsenoside Rg1 and Rb1, as well as minor ginsenosides like ginsenoside Rg3 and CK, as illustrated in Figure 1. Notably, the major ginsenoside Rg1 was not detected in HME-BG, and the content of Rb1 decreased by approximately 32% compared to BG. In contrast, the minor ginsenoside Rg3 increased by about 330%, and CK showed an increase of approximately 450% compared to BG. These results indicate that the major ginsenosides (Rg1, Rb1) in BG were structurally converted into minor ginsenosides (Rg3, CK) through HME treatment, resulting in a significant increase in their content. Additionally, the chemical structures of each component are illustrated in Figure 2.

These findings suggest that the structural modification of ginsenosides induced by the high-temperature and high-pressure conditions of HME is consistent with the results of previous studies. Earlier research has shown that major ginsenosides, such as Rg1 and Rb1, undergo structural transformation into minor ginsenosides, like Rg3 and CK. This transformation is attributed to the hydrolysis of sugar moieties at the C-3, C-6, and C-20 positions, followed by dehydration at C-20 during the heating process used in the production of red and black ginseng [11].

### 3.2. Particle Size, Polydispersity Index, and Zeta Potential

The particle size, polydispersity index (PDI), and zeta potential (ZP) measurements for BG and HME-BG are summarized in Table 2. It is well-established that reducing particle size can enhance technical characteristics and influence physicochemical properties such as surface area, solubility, and fluidity [22]. PDI is used to assess the breadth of particle size distribution, with a value of 0.7 or higher indicating a broad distribution, while lower values suggest a narrower and more uniform particle size distribution [23]. ZP is an indicator of particle stability, reflecting the electrical repulsion between particles; a higher absolute ZP value signifies greater dispersion stability [24].

The results showed that the particle size of HME-BG was reduced by approximately 34% compared to BG. This reduction in particle size is expected to increase the surface area per unit mass, thereby improving the solubility and release of active compounds, such as ginsenosides. Additionally, smaller particle sizes are more likely to be absorbed by cells and tissues, potentially enhancing absorption efficiency and bioavailability in intestinal tissues [25,26]. Furthermore, the PDI significantly improved from approximately 0.71 to 0.38, indicating a more uniform particle size distribution. The zeta potential remained relatively stable at around −20 mV for both BG and HME-BG, suggesting consistent particle stability.

### 3.3. X-ray Diffraction Data

**Figure 3:**
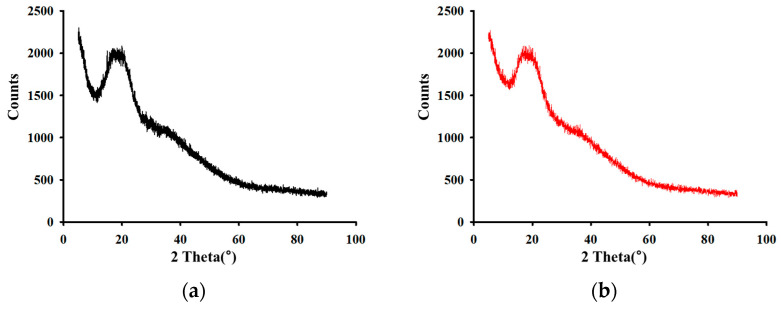
X-ray diffraction analysis: (**a**) Black ginseng powder (BG); (**b**) HME-treated black ginseng powder (HME-BG).

The crystallinity of BG and HME-BG was characterized using X-ray diffraction (XRD) analysis [27], as shown in Figure 3. Both samples exhibited broad and diffuse peaks, indicating their predominantly amorphous nature. However, the peaks in Figure 3a were more intense, suggesting that BG contains relatively larger particle sizes and a higher degree of crystallinity compared to HME-BG. This observation implies a reduction in particle size and crystallinity in BG following HME treatment. Previous studies have shown that amorphous particles exhibit a higher dissolution rate and better dispersion behavior compared to crystalline particles, leading to increased oral bioavailability [28]. Therefore, we anticipate that reducing the crystallinity of HME-treated black ginseng (HME-BG) will enhance its bioavailability.

### 3.4. Fourier-Transform Infrared Analysis and Thermogravimetric Analysis

**Figure 4:**
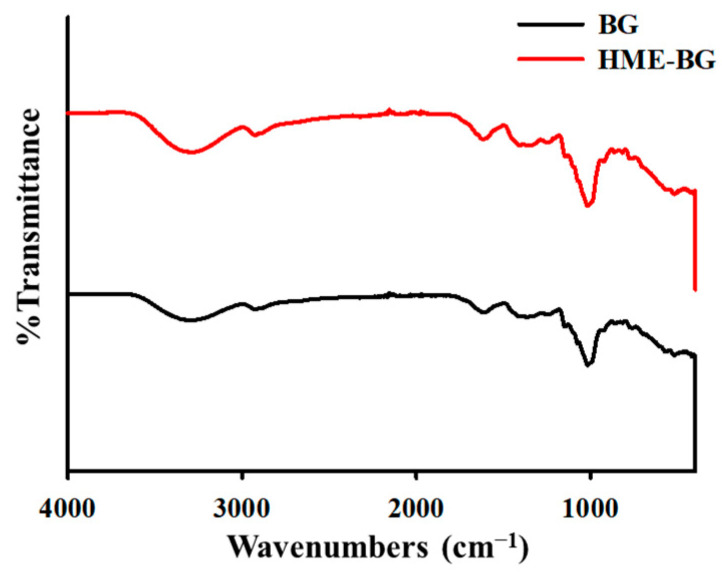
Fourier-transform infrared analysis.

BG and HME-BG were characterized by Fourier-transform infrared (FT-IR) spectroscopy within the range of 4000–400 cm^−1^ (Figure 4). The two spectra exhibited very similar profiles, with five common peaks observed around 3290, 2920, 1620, 1360, and 1010 cm^−1^. The band at 3290 cm^−1^ corresponds to the C-H stretching vibration, while the peak at 2920 cm^−1^ is associated with the stretching vibration of the –CH_2_– group. The signal at 1620 cm^−1^ can be attributed to the stretching vibration of carbonyl groups present in volatile oils and other C=O- containing compounds [29]. The dip around 1360 cm^−1^ is linked to the symmetric stretching vibration of the COO^−^ group. In the spectral range of 1150–911 cm^−1^, characteristic bands of many C-O-C groups are observed, with the strong band at 1010 cm^−1^ generally related to the C-O bond vibration of the hydroxyl group in alcohols [30]. It was noted that the intensities of all these bands decreased in HME-BG compared to BG, suggesting that the major ginsenosides were converted to minor ginsenosides during high-temperature HME treatment, likely through a heat-driven deglycosylation process [10,31].

**Figure 5:**
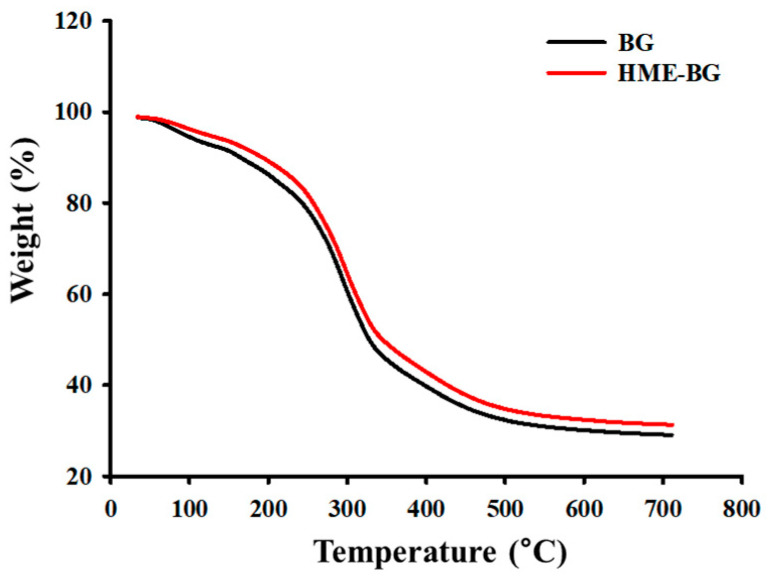
Thermogravimetric analysis.

The thermal behavior of BG and HME-BG was examined using thermogravimetric analysis (TGA), as illustrated in Figure 5. TGA is a technique used to measure weight changes in a sample as its temperature is varied over time in a controlled environment, providing insights into the composition and decomposition processes of the samples. The comparison of the TGA results for BG and HME-BG revealed no significant differences between the two samples, indicating that the thermal conditions applied during the HME treatment did not alter the thermal properties of the samples.

### 3.5. Limitations of This Study and Directions for Future Research

This study successfully increased the content of minor ginsenosides, such as Rg3 and CK, in black ginseng through hot melt extrusion (HME), a high-temperature and high-pressure processing technique. Numerous previous efforts to enhance ginsenoside content and convert major ginsenosides into minor ones have employed physical (heating, drying, microwave), chemical (acid–base hydrolysis), and biological (enzymatic, microbial) methods, yielding significant results. However, these methods often have drawbacks, including the use of organic solvents, high costs, complex processes, environmental pollution, and long processing times [13,14,15]. In contrast, HME does not require organic solvents; is easily optimized, eco-friendly, and cost-effective; and involves a streamlined, automated process that shortens processing time without producing by-products or carbon emissions.

This study confirmed an increase in minor ginsenosides and improvements in the physicochemical properties of the particles, including particle size, dispersion, and crystallinity. These results suggest that HME-treated black ginseng (HME-BG) may enhance biological activity, such as bioavailability. Future in vitro and in vivo studies will be needed to validate these potential benefits.

Furthermore, if HME-BG demonstrates improved minor ginsenoside content and biological activity compared to existing products like dietary supplements, functional foods, and pharmaceuticals derived from ginseng, red ginseng, and black ginseng, it is expected to have broad applications across various industries. This advancement could offer a significant competitive advantage over current products.

## 4. Conclusions

This study demonstrated that HME treatment of black ginseng led to an increase in specific minor ginsenosides and a reduction in particle size and dispersion. Minor ginsenosides are known for their enhanced pharmacological efficacy and bioavailability compared to major ginsenosides. Furthermore, the HME process improved the particle size, PDI, and crystallinity of black ginseng powder without compromising its chemical structure or thermal properties. These findings indicate that HME is a highly effective method for processing black ginseng, offering potential for high-value-added applications in the future.

## Figures and Tables

**Table 1:** The HPLC conditions for analysis of ginsenosides.

**HPLC** **Analysis** **Conditions**	Column	YMC Triart C18 (5 μm, 12 nm) 250 × 4.6 mm	
	Detector	Diode Array Detector (DAD)	
	Oven	40 °C	
	Solvent A	Water	
	Solvent B	Acetonitrile (ACN)	
	Flow rate	1.0 mL/min	
	Injection volume	10 μL	
	Gradient elution system		
	Time (m)	%A	%B
	Initial	95	5
	35 m	65	35
	40 m	20	80
	72 m	95	5
	90 m	95	5

**Table 2:** Particle size, polydispersity index (PDI), and zeta potential (ZP) of black ginseng powder (BG) and HME-treated black ginseng powder (HME-BG).

	Particle Size (nm)	PDI (Index)	ZP (mV)
BG	864.7 ± 57.30	0.710 ± 0.056	−21.21 ± 1.02
HME-BG	599.2 ± 41.12	0.380 ± 0.019	−18.88 ± 0.43

## Data Availability

Data are contained within the article.
